# Mixed-methods research: A tutorial for speech-language therapists and audiologists in South Africa

**DOI:** 10.4102/sajcd.v65i1.573

**Published:** 2018-07-12

**Authors:** Anna-Marie Wium, Brenda Louw

**Affiliations:** 1 Discipline Speech-Language Pathology and Audiology, Sefako Makgatho Health Sciences University, South Africa; 2 Department of Audiology and Speech-Language Pathology, College of Clinical and Rehabilitative Health Sciences, East Tennessee State University, United States

## Abstract

**Background:** Mixed-methods research (MMR) offers much to healthcare professions on clinical and research levels. Speech-language therapists and audiologists work in both educational and health settings where they deal with real-world problems. Through the nature of their work, they are confronted with multifaceted questions arising from their efforts to provide evidence-based services to individuals of all ages with communication disorders. MMR methods research is eminently suited to addressing such questions.

**Objective:** The aim of this tutorial is to increase awareness of the value of MMR, especially for readers less familiar with this research approach.

**Method:** A literature review was conducted to provide an overview of the key issues in MMR. The tutorial discusses the various issues to be considered in the critical appraisal of MMR, followed by an explanation of the process of conducting MMR. A critical review describes the strengths and challenges in MMR.

**Results:** MMR is less commonly used or published in the fields of speech-language therapy and audiology.

**Conclusion:** Researchers working in teams can draw on the strengths of different disciples and their research approaches. Such collaborative enterprises will contribute to capacity building. Researchers, SLTs and audiologists are encouraged to make use of MMR to address the complex research issues in the multicultural, multifaceted South African context. MMR makes an important contribution to the understanding of individuals with communication disorders, and in turn, researchers in the two disciplinary fields of speech-language therapy and audiology can contribute to the development of this research approach. MMR is well suited to the complexity of South African contexts and its populations, as it can provide multiple perspectives of a topic.

## Introduction

Traditionally, the emphasis in research in the fields of speech-language therapy and Audiology has been on quantitative research, in large part as a result of the medical model that dominated the field for many years. The qualitative research paradigm started to gain popularity in the late 1980s shifting to a more biopsychosocial approach as propagated by the World Health Organization (2001). This shift was brought about by research becoming more person-centred, as well as by the impact of qualitative research in fields such as education, social sciences and psychology. Mixed-methods research (MMR) is a more recent development in the research arena and can make an important contribution to the understanding of individuals with communication disorders. Research in the disciplines of speech-language therapy and audiology continues to evolve as methodological developments occur, which impact researchers and clinicians alike.

Johnson, Onwuegbuzie and Turner (2007) discuss many definitions for MMR, but for the purpose of this discussion the explanation by Creswell and Plano Clark (2011) was deemed to be the most explanatory. Mixed-methods research is a research approach or methodology focusing on:

- research questions that call for real-life contextual understanding, multilevel perspectives and cultural influences
- employs rigorous quantitative research assessing the magnitude and frequency of constructs and rigorous qualitative research exploring the meaning and understanding of constructs
- utilises multiple methods (e.g. intervention trials and in-depth interviews)
- intentionally integrates or combines these methods to draw on the strengths of each and framing the investigation within philosophical and theoretical positions.

Yin (2006) described high-quality MMR as studies where the mixing occurs from formulating the research questions right through to the interpretation of findings. Not all MMR is reported as such as many studies may include only a small component from one of the methods (e.g. a few open-ended questions in a mainly closed-ended questionnaire to enhance the findings). The assumption in MMR is that the use of quantitative and qualitative approaches in combination provides a better understanding of research problems than either approach alone (Creswell & Plano Clark, 2007). Mixed-methods research collects and analyses data, integrates the findings and draws inferences using both qualitative and quantitative approaches in a single study or programme of inquiry (Creswell, Klassen, Plano Clark, & Clegg Smith, 2011).

Mixed-methods research is a relatively new approach in the fields of speech-language therapy and audiology and has been under-represented in these disciplines as compared, to mono-method approaches. Suleman and Hopper (2014) reported that less than 1.2% of studies published in the four most prominent speech-language therapy and audiology journals in the United States and Canada during the period 2007–2011, used an MMR approach. An electronic search of the term *mixed-method* or *quantitative and qualitative* through the AOSIS search engine in the *South African Journal of Communication Disorders* over the past 10 years showed that 12 articles have been published from MMR studies, of which eight articles reported on both quantitative and qualitative findings, and four articles reported findings from single methods, but stated that it was part of a larger MMR project and/or study through findings from an electronic search may not be as exact as a paper search, it does give an indication of a scarcity of such research in the field.

There appears to be some uncertainty as to what can be considered as MMR and what such studies entail. Many studies that incorporate both quantitative and qualitative methods for data collection do not include the term MMR in the title of the research (refer to Table 1). It is also noted that when such a term is included in the title, and both quantitative and qualitative methods are included in the research, the researchers rarely explain how the two methods have been integrated (Hashemi & Babaii, 2013), or where in the research process the mixing did occur (Azul, Arnold, & Neushaefer-Rube, 2018).

**TABLE 1:** Mixed-methods research articles published in the South African Journal of Communication Disorders (2008–2018).

Number	Publication	Report: single or both methods
1	Wium, A. M., Louw, B., & Eloff, I. (2010). Speech-language therapists supporting foundation-phase teachers with literacy and numeracy in a rural and township context. *South African Journal of Communication Disorders, 57*(1), 14–22.	Quantitative and qualitative (QUAN+QUAL)
2	Wium, A. M., Louw, B., & Eloff, I. (2011). Evaluation of a programme to support foundation-phase educators to facilitate literacy. *South African Journal of Communication Disorders, 58*(2), 72–78.	Qualitative
3	Wium, A. M., & Louw, B. (2011). Teacher support – An exploration of how foundation-phase teachers facilitate language skills. *South African Journal of Communication Disorders, 58*, 86–94.	Qualitative
4	Wium, A. M., & Louw, B. (2012). Continued professional development of teachers to facilitate language used in numeracy and mathematics. *South African Journal of Communication Disorders, 59*, 8–15.	Quantitative and qualitative (QUAN+QUAL)
5	Teixeira, L., & Joubert, K. (2014). Availability of audiological equipment and protocols for paediatric assessment and hearing aid fitting in Gauteng, South Africa. *South African Journal of Communication Disorders, 61*(1), 8.	Quantitative and qualitative (QUAN+qual)
6	Navsaria, I., Pascoe, M., & Kathard, H. (2011). ‘It’s not just the learner, it’s the system!’ Teachers’ perspectives on written language difficulties: Implications for speech-language therapy. *South African Journal of Communication Disorders, 58*, 95–104.	Qualitative
7	Mdlalo, T., Flack, P. S., & Joubert, R. (2016). Are South African speech-language therapists adequately equipped to assess English Additional Language (EAL) speakers who are from an indigenous linguistic and cultural background? A profile and exploration of the current situation. *South African Journal of Communication Disorders, 63*(1).	Quantitative and qualitative (QUAN→QUAL+quan)
8	Lundie, M., Erasmus, Z., Zsilavecz, U., & Van der Linde, J. (2014). Compilation of a preliminary checklist for the differential diagnosis of neurogenic stuttering. *South African Journal of Communication Disorders, 61*(1), 10.	Quantitative and qualitative (QUAN→qual)
9	Wium, A. M., & Gerber, B. (2016). Ototoxicity management: An investigation into doctors’ knowledge and practices, and the roles of audiologists in a tertiary hospital. *South African Journal of Communication Disorders, 63*(1).	Quantitative and qualitative (QUAN+qual)
10	Schütte, U. (2016). Culturally sensitive adaptation of the concept of relational communication therapy as a support to language development: An exploratory study in collaboration with a Tanzanian orphanage. *South African Journal of Communication Disorders, 63*(1), a166.	Quantitative
11	Andrews, M., & Pillay, M. (2017). Poor consistency in evaluating South African adults with neurogenic dysphagia. *South African Journal of Communication Disorders, 64*(1).	Quantitative and qualitative (QUAN+qual)
12	Abdoola, F., Flack, P. S., & Karrim, S. B. (2017). Facilitating pragmatic skills through role-play in learners with language-learning disability. *South African Journal of Communication Disorders, 64*(1), a187.	Quantitative and qualitative (QUAN+qual)

Researchers also tend to report results obtained from MMR as a single method (refer to Table 1), as if two separate studies were conducted within one topic. The reason to do this may be attributed to the length of such studies that makes it difficult to keep to word count restrictions stipulated by most journals. Another reason for confusion is that studies are referred to as MMR when questionnaires are used with mainly closed-ended questions but with a single open-ended question included at the end (Bryman, Becker, & Sempik, 2008).

Considering these aforementioned reasons for confusion, some clarification is required for researchers and clinicians in terms of MMR concepts and methods. The aim of this tutorial is to provide researchers, speech-language therapists and audiologists with an overview of MMR to serve as alternative to the traditional quantitative and qualitative research approaches in their pursuit of evidence-based practice (EBP). Based on a comprehensive and in-depth literature review, the tutorial firstly provides a background that explains MMR in terms of the philosophy and its relationship to EBP. Next, the definition and clarification of concepts, as well as the characteristics of MMR are explained. The article discusses the various issues to be considered in the critical appraisal of MMR, followed by a description of the process of conducting MMR. A critical review of MMR describes the advantages, as well as the controversies and challenges in MMR. Lastly, a conclusion highlights the importance of MMR for the disciplines of speech-language therapy and audiology.

## Background

As a methodology, MMR involves philosophical assumptions that guide the direction of the collection and analysis of data and the mixture of qualitative and quantitative data in a single study or series of studies.

### Philosophical underpinnings of mixed-methods research

New methodologies evolved that combine and integrate quantitative and qualitative research approaches, which open up more possibilities in answering specific research questions (Glogowska, 2011). In studies where specific health issues of a population need to be assessed, it calls for counting and measuring or comparing, which rely on quantitative inquiries. Qualitative approaches are in turn more suitable whenever explanations for certain phenomena are called for (e.g. when clients’ understanding of their problems needs to be determined, their perceptions of treatments are sought or the determination of how services have been delivered).

When considering the nature of the research, there are definite differences between quantitative, qualitative and MMR approaches (Creswell & Plano Clark, 2007; Tashakkori & Teddlie, 2010). In terms of the nature of reasoning (ontology), quantitative research is deductive, whereas qualitative research is adductive. Between these two ontologies, MMR is inductive. When considering the nature of reality (axiology), quantitative research has a single reality view, as opposed to qualitative research where multiple views of reality exist. Mixed-methods research draws from both these realities as it includes both single and multiple realities.

In terms of the epistemology, the nature of knowing in quantitative research is objective, as opposed to qualitative research, where the nature of knowing is subjective. Once again MMR takes the middle ground as the nature of knowing is intersubjective.

The role of values in the interpretation of results also differs, as quantitative researchers strive to be unbiased and take precautions to avoid bias, which is different from qualitative research where the researchers are integrally part of the research and, therefore, are fundamentally biased. In MMR, the interpretation of findings is both biased and unbiased as both quantitative and qualitative methods are used. However, in MMR, care is taken to limit bias by putting specific measures in place. In the interpretation of results, quantitative results can be generalised because of the sample size and selection methods being used, which is not the case in qualitative research where smaller samples are typically used which makes the findings more context specific. In MMR, the findings are transferrable to similar contexts and population groups because thick descriptions are used in the qualitative strand and also because a sufficient sample size is being used in the quantitative strand. The three research approaches also differ in terms of causality: in quantitative research the cause results in effect, whereas in qualitative research the cause cannot be isolated from the effect. In MMR, causality cannot be determined. It can, therefore, be concluded that because both the quantitative and qualitative approaches are included in MMR, it has a unique underlying philosophy that is based in the common ground between these two methodologies.

In the case of MMR, the claim of knowledge (philosophical assumption) often is pragmatic because the rigid interpretations of methodologies have begun to fade (Onwuegbuzie & Dickinson, 2007). Christ (2013) shows the relevance of a critical realist stance and how these two approaches are very similar in practical terms. Mertens (2011) provides insights into a more transformative paradigm for whenever researchers show concern for priority on social justice and the furtherance of human rights. Other alternatives include the dialectical position (Johnson et al., 2007) or the critical interpretive view (Denzin & Lincoln, 2005). These philosophical positions need not be compared as it is evident that the mixing of methods could contribute to a better understanding of the research question (Hashemi & Babaii, 2013). In short, mixed research acknowledges a meta-paradigmatic existence.

Research in speech-language therapy and audiology aims to not only accumulate knowledge concerning communication and its disorders but also consider the therapeutic issues that could improve the quality of life of individuals with communication disorders. Such knowledge should inform practice. Evidence-based practice emerged as an important principle in the delivery of speech-language therapy and audiology services in the past decade (American Speech-Language-Hearing Association, 2005).

### Evidence-based practice and implementation science

**FIGURE 1:**
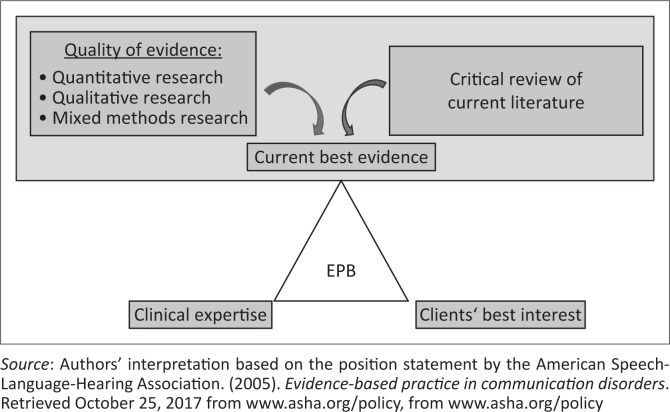
Evidence-based practice.

Evidence-based practice involves the integration of best current research evidence, clinical expertise and the needs, abilities, values, preferences and interests of clients and their families in making clinical decisions to provide high-quality services and, therefore, EBP is central to the disciplines of speech-language therapy and audiology (American Speech-Language-Hearing-Association, 2005). The trilateral principles forming the basis for EBP (American Speech-Language-Hearing Association, 2005) are illustrated in Figure 1, with emphasis on the research evidence.

However, a gap often exists in the application of knowledge to practice, which opens up the field of implementation science (Olswang & Prelock, 2015), which focuses on research methods that facilitate the systematic application of research to practice in both the health and education sectors. Such research identifies, defines and evaluates the strategies used in the treatment of communication disorders and requires the use of both quantitative and qualitative data (Olswang & Prelock, 2015). Implementation science and EBP have led to an increased need for applied clinical research in the speech-language therapy and audiology disciplines to answer multiple questions that are related to the ‘what?’, ‘why?’ and ‘how?’ of assessment and intervention, for which a variety of quantitative and qualitative data or both are required.

Systematic reviews were developed to support EBP and can be performed on MMR by analysing both quantitative and qualitative research evidence. In this case, it is necessary for data analyses to be carried out separately in order to ensure the integrity of the research. Mixed-method systematic reviews are suitable to understand the barriers and facilitators to the implementation of certain interventions, to assess cost-effectiveness and to address the core competencies of practitioners. These systematic reviews add important value to traditional systematic reviews as the inclusion of qualitative studies provides a greater understanding of the appropriateness of an intervention beyond documentation of its effectiveness (Orlikoff, Schiavetti, & Metz, 2015).

Mixed-methods research provides the opportunity to seek answers to complex clinical questions and to explore evidence from both quantitative and qualitative perspectives in the pursuit of improving outcomes. It is important for speech-language therapists and audiologists to be cognisant of the MMR approach and how it can be applied in EBP.

## Demystifying mixed-methods research

Mixed-methods research is often criticised for a lack of a common definition, confusing terminology and limited guidelines for when it is suitable to select MMR methods (Creswell, 2013). The question arises as to when to select a MMR approach?

### When is mixed-methods research suitable?

The most probable answers to this question are when either the quantitative or the qualitative methods by itself appear to be inadequate to create a complete understanding of the research problem, or to develop multiple perspectives of a phenomenon, for example, when quantitative outcome measures are enhanced or explained by using qualitative data. On the other hand, a qualitative exploration, for example, a focus group discussion prior to the development of an instrument or measurement tool, may inform the contents and approach thereof.

Creswell and Plano Clark (2011) identified further reasons for using MMR, such as when information needs to be contextualised and it may be necessary to obtain a macro picture of a system (e.g. a school or hospital). It could be that the researcher might want to develop a complementary picture; to compare, validate or triangulate results; to provide an illustration of context and trends; or to examine process or experiences along with outcomes. Mixed-methods research may also be useful when one database needs to be built on another, or when one method is failing and the second method can be used to rescue the study. According to Glogowska (2011), MMR is a good option when conducting research on marginalised groups as it provides them with a voice when research is conducted concerning them, which in effect is empowering them. Novice researchers need to understand the terms and concepts, as well as the characteristics and notations used in MMR before applying the approach.

### Clarification of terms and concepts in mixed-methods research

Several terms are used to describe the nature and extent of mixing methods (e.g. mixing, combining and integrating), and these are often used interchangeably (Creswell, Plano Clark, Gutmann, & Hanson, 2003; Teddlie & Tashakkori, 2003). For clarity, such multiple method designs can be described as either mixed-methods designs or multimethod research. These two concepts should not be confused. In multimethod research, the research question is answered by using two data collection procedures or methods, each from the same quantitative and qualitative traditions and is known as either a multimethod qualitative study or a multimethod quantitative study. Mixed-method designs can either be MMR or mixed-model research. For instance:

- Mixed-methods research is where the mixing occurs in the methods stage of the study and it can be on a continuum from simple to complex (Onwuegbuzie & Johnson, 2006). The qualitative and quantitative data collection and analysis are performed in either parallel or sequential phases. The term *combining methods* refers to a situation where one method paves the way for the other, or follows the first method in an effort to help or assist towards the planned outcomes or intention of the research. In this case, the two approaches are not weighted equally. *Integrating methods* is used when the methods are kept separate at the paradigm level, but are interlinked with each other as it looks at the same phenomenon from two different perspectives to create a more comprehensive view of the phenomenon (Woolley, 2009). The integration can occur any time from the very beginning or towards the end when the results are written up (Glogowska, 2011). It may be that sampling and data collection are carried out separately, but that the integration occurs during data analysis, interpretation or theory generation. The study by Abdoola, Flack and Karrim (2017) that facilitated pragmatic skills through role-play in learners with language-learning disability kept the two sets of data separate and only integrated them at the reporting level through a weaving approach.
- Mixed-model research is where the mixing occurs in all stages of the study (the research question, methods, data collection and analysis and inference processes).

### Mixed-methods research: Characteristics and notations

There are definite differences that distinguish MMR from mono-method designs (Garuth, 2013). In MMR, the researchers should offer a rationale for using both quantitative and qualitative methods in the research and also clearly state that both quantitative and qualitative data have been collected. There needs to be a clear statement that indicates which method carries more weight and what the sequence of data collection procedures is (i.e. sequential or concurrent). The analysis of data is related to a specific design type (e.g. convergent, explanatory, exploratory or embedded design), although not limited to these. In MMR, a diagram is provided that shows the procedures used in the study (Creswell and Plano Clarke 2011),

Morse (1991) provided mixed-methods notations that are useful for representing the different designs which are referred to in the literature. The use of upper case refers to emphasis (i.e. the primary or dominant method), whereas the use of lower case refers to lower emphasis, priority or dominance:

- QUAN or quan refers to quantitative data
- QUAL or qual refers to qualitative data
- MM refers to mixed-methods
- → data collected sequentially
- + data collected simultaneously
- = converged data collection
- ( ) one method embedded in the other.

Knowledge of the notation system used in MMR makes it easier to read and to critically appraise such studies.

## Critical appraisal of mixed-methods research

Speech-language therapists and audiologists are required to critically appraise research before selecting the findings for making EBP decisions. Many research studies include both quantitative and qualitative data collection and analysis methods. The quality of research depends on how well the accepted form for both qualitative and quantitative research designs is followed (Garuth, 2013). The following steps and guidelines are proposed for readers when conducting a critical appraisal of MMR.

### Initial identification

It is recommended that the process be initiated by performing a general screening procedure that involves scanning the abstract and methodology sections of an article to determine where in the research process the mixing occurred (e.g. at the research questions stage, the sampling stage, the data collection or analyses). It is important to search for key words that refer to terms, for example, mixed- or multimethods, or qualitative, quantitative, triangulation, integrating methods, and combining methods. Once the initial scanning process has been completed it is necessary to conduct a rigorous content analysis to look for rich descriptions of the content, particularly on how the mixing and/or combining was performed. At this stage of the appraisal, it is necessary to identify the design of the study, the type of sampling and the data collection and analysis. Next, one has to consider issues of validity and whether there are any signs of using meta-inferences that could indicate inference quality (Tashakkori & Teddlie, 2010).

### Identification of the mixed-methods research designs

The second step in the critical appraisal of MMR is to identify the research design and to ensure that a pattern is used throughout the research. The specific mixed-methods design is determined by two main factors (Tashakkori & Teddlie, 1998; Creswell, 2003; Onwuegbuzie & Dickinson, 2007): (1) priority of weight, where equal weight can be allocated to both quantitative and qualitative aspects, or alternatively, different weights can be given; (2) order of data collection, which refers to the order in which qualitative and quantitative data can be collected. Creswell et al. (2011) classified MMR designs as not only concurrent or sequential, but also transformative.

***Concurrent designs:*** are when quantitative and qualitative data are collected at the same time. Such designs are referred to as simultaneous, concurrent, convergent, or parallel designs (Irwin, Pannbacker, & Lass, 2014). The two subtypes are:

- Concurrent Triangulation Designs: *Concurrent (convergent) procedures* are used to converge quantitative and qualitative data in order to provide a comprehensive analysis of the problem. In this case, the qualitative and quantitative data are collected simultaneously. The results are then integrated in the final interpretation. Merging of QUAN and QUAL results occurs during the analysis or interpretation to provide an integrated conclusion and involves comparing, contrasting and synthesising the two strands (Creswell & Plano Clark, 2011). For example, Overby, Carrell and Bernthal (2007) investigated second-grade teachers’ perceptions of the academic, social and behavioural competence of second-grade students with speech-sound disorders (SSDs) by converging quantitative results with qualitative detail using a concurrent MMR design.
- Concurrent designs may also comprise data being collected by using both open-ended and close-ended questions with a survey instrument (Creswell & Plano Clark, 2011), such as was employed by Teixeira and Joubert (2014) in their descriptive, cross-sectional survey to describe the availability of clinical audiological equipment and protocols used in Gauteng. Bedwinek, Kummer, Rice and Grames (2010) explored preschool and school-based speech-language therapists’ assessment and treatment practices of children with cleft lip and palate to determine continuing education needs in this area. Quantitative data were collected through a survey using Likert-type scales and responses analysed descriptively. The qualitative data were collected concurrently using open-ended questions that were designed to be complementary to quantitative questions. Thematic analyses were used to examine and interpret the responses to the open-ended questions. A similar example can be found in a study that described the poor consistency in evaluating South African adults with neurogenic dysphagia, where the small qualitative component collected concurrently in a mainly closed-ended questionnaire was subjected to textual analyses and themes were coded into numerical values (Andrews & Pillay, 2017).

***Concurrent embedded design:*** In concurrent (convergent) designs, one form of data collection is often *nested* within another (larger) data collection procedure to analyse different questions or levels of units Creswell et al. (2011) for example: QUAL + Quan; QUAN + Qual. The primary purpose is to enhance the traditional QUAL or QUAN design (Creswell, 2013). In this case, the two components have unequal weighting and the timing could be either concurrent or sequential. These designs can be configured as either that the QUAN is emphasised and the qual is supplemental, or vice versa.

Embedded designs have two research questions, which should be answered separately. The supplemental data set should answer the second research question. Danzak (2011a), for example, first reported the QUAN component of her research, which was on writing samples from English language learners that were analysed for linguistic complexity to establish a difference between English and Spanish. In the second article based on the qualitative component, the author reported on the analyses of English language learners’ journal entries to determine the impact of literacy experiences as bilingual writers (Danzak, 2011b). Similarly, Wium (2010) developed a support programme for foundation-phase teachers to facilitate literacy and numeracy skills, which was evaluated using a variety of quantitative and qualitative methods. QUAN data were collected with questionnaires from 96 participants to determine how the programme was implemented in two different contexts, together with portfolio assessments. In an embedded design, qualitative data were collected from six focus groups (Qual) consisting of six participants each who were selected from the larger group, as well as journal entries to determine how the participants experienced the programme. As the primary and secondary data in these designs addressed distinct questions, the results were collected separately and were reported on separately, which were different but related. The integration occurred when qualitative data were transformed to be compared with quantitative data in table format to answer the specific research questions (Wium, Louw, & Eloff, 2010). The quantification of qualitative data for comparison with quantitative data has been described by Creswell (2003) and is considered as an acceptable method of data integration in MMR, although this practice may be considered as controversial by some qualitative researchers.

***Sequential designs***: These types of designs lend themselves to studies where the QUAN and QUAL components are not equal in weight and data are collected sequentially in phases, where the first phase informs the second phase. These designs provide a summary of both sets of results with a discussion on how the second phase confirmed or expanded on the first phase (Creswell & Plano Clark, 2011). The mixing occurs in the interpretation. The two types of sequential designs are the following:

- Sequential Explanatory Design: an alternative is the *explanatory design (QUAN* → *qual)*, where one would start off with a quantitative method to test theories or concepts, and then to follow up with qualitative methods with a small group of participants to explore the issues further (Creswell & Plano Clark, 2011). An example would be where participants with hearing loss are asked to rate their conversational abilities before and after an aural rehabilitation programme (QUAN). Some of these participants will then be interviewed individually afterwards to discuss the reasons for their ratings (qual) (Suleman & Hopper, 2014).
- Sequential Exploratory Design: the qualitative strand helps to develop or inform the quantitative strand and to connect the data between the two phases (e.g. in instrument design, theory building or testing). The emphasis usually is on QUAL, but can be QUAN, or it can be equal. The mixing occurs in the interpretation of the results – (QUAL → quan). The QUAL part of the study will provide information to uncover variables of interest. The second phase would be where the quan phase is used to develop the tool from the results obtained in the first phase. A focus group in a school could explore how teachers determine perceived barriers to receive speech-language services at their schools (qual), and then these ideas will be used to conduct a large-scale survey in a district by asking the participants to rate the impact of predetermined barriers (QUAN) (Suleman & Hopper, 2014). Another example could be found in a study by Langevin, Packman and Onslow (2009), where the researchers determined whether specific characteristics of participants’ stuttering patterns elicited negative peer responses by firstly transcribing play sessions (QUAL) and then went on to analyse stuttering behaviours, durations of stutters and judgements of the meaningfulness of peer-directed stuttered utterances (QUAL → quan).

### Research sampling

The third step in the critical appraisal of MMR is to review the sampling process. Collins, Onwuegbuzie and Sutton (2006) described a model for MMR sampling, which categorise sampling designs according to the time orientation of the components and the relationship of the qualitative and quantitative samples. In terms of the time orientation, the sampling can be carried out either concurrently or sequentially. The relationship between these two types of samples can be one of the following:

- Identical sampling is where the qualitative and quantitative samples include the same participants.
- Parallel sampling design is where different qualitative and quantitative samples are drawn from the same population. There is parallel use for probability and purposive strategies, either concurrently or sequentially. Examples are where one set may be a subset of the other, or where both studies use the same total sample.
- Nested sampling design is where the participants from one component of the investigation represent a subset of those who were included in another phase of the study.
- Multilevel sampling makes use of sample sets from different populations at different levels of the study. In this case, probability and purposive sampling techniques are used at different levels of analysis (e.g. therapists and clients).

### Data collection

The fourth step in the critical appraisal of MMR is to evaluate the data collection. Data are collected simultaneously (concurrently) or sequentially (Creswell & Plano Clark, 2011). An example of a concurrent design in speech-language therapy was provided by Bedwinek et al. (2010), who performed a national survey using MMR to determine the training needs of preschool and school-based speech-language therapists regarding children with cleft lip or cleft palate. These authors (*ibid*) collected quantitative data using a Likert-type scale and concurrently also collected qualitative data from open-ended questions that were complementary to the quantitative questions in the same questionnaire.

In a sequential study, Overby et al. (2007) collected quantitative data to determine the extent of differences between teachers’ perceptions of students with SSDs and those with typically developing speech by means of audiotaped sentences as stimuli that had to be rated. This phase was followed by collecting qualitative data through open-ended questions to discover insights into the characteristics of any similarities or differences. Another example of a sequential study is that of Langevin et al. (2009) who explored the social impact of stuttering of preschoolers in free-play by investigating peer responses to stuttered utterances. Four free-play sessions were audio- and videotaped and participants’ intelligible utterances were transcribed. Inter-observer reliability was obtained from independent research assistants who were speech-language therapists by randomly selecting different subsets of data for each reliability measure to confirm the integrity of the research.

In a nested MMR design, Bryman et al. (2008; Bryman, 2006) investigated social policy researchers in the United Kingdom. They made use of an e-survey to investigate views regarding quality. In a nested MMR design, the researchers used semi-structured interviews (per telephone) with a small number of participants from the original sample. The use of open-ended questions allowed the participants to express opinions that could not be covered with the closed-ended questions in the e-survey. The qualitative component allowed several issues to be explored in greater depth, which enhanced the survey findings.

### Data analysis and integration

It is essential that one evaluates or assesses which data analyses were employed when critically appraising MMR. Various data analyses methods can be used in MMR. Quantitative data analysis is either descriptive and/or inferential, whereas qualitative data analysis is carried out descriptively and through thematic analyses of text or image. Overby et al. (2007) made use of descriptive statistics to show differences in the means of all dependent variables, as well as multiple analyses of variance to disclose statistical differences between intelligibility levels. The study by Bryman et al. (2008) used descriptive statistics with frequencies from the e-survey with contingency table analysis of specific quality criteria. The qualitative data from telephone interviews were recorded, transcribed and analysed thematically.

In the Langevin et al. (2009) study, the quantitative and qualitative results were used to answer different components of the research. Data were analysed separately for each research question. Peer responses to stuttered utterances were recorded. The duration of stuttering behaviours was measured in utterances that elicited negative responses from peers by the use of, ‘*The Stuttering Severity Instrument for Children and Adults* 3rd Edition’ (Riley, 1994). In addition, responses were coded as either neutral or positive (no adverse attention by peer) or negative, which in turn was behaviourally described and labelled. The meaningfulness of all peer-directed stuttered utterances was coded and inter-coder reliability was obtained. In all these aforementioned studies, the quantitative and qualitative analyses were carried out separately. None of the aforementioned studies or researchers provided a clear explanation of how the qualitative and quantitative methods were integrated.

Once the initial quantitative and qualitative data analyses have been completed, the MMR analysis should be carried out. Creswell (2003) described four strategies for data analysis in MMR, which include data transformation, exploration of articles, instrument development and examination on multiple levels, although operational definitions that describe objectively measurable indicators for such an analysis are not specifically described. Tashakkori and Teddlie (2010) in turn described integrative efficacy as how well the inferences made in each strand of the MMR can be integrated into a theoretically consistent meta-inference (Tashakkori & Teddlie, 2010). These authors (*ibid*) proposed three methods for integrating multiple forms of data:

- *Merging data:* qualitative data (texts or images) can be combined with quantitative data (numeric information). When merging data, the process of integration occurs when qualitative and quantitative data are merged to obtain the results. Merging involves equal emphasis on both strands of the research as the results are compared, contrasted or synthesised (Suleman & Hopper, 2014). The method of merging is to report qualitative and quantitative results together in a discussion section, for example, where the quantitative statistical results are reported first, followed by quotes or themes that either support or refute the quantitative results. Merging can also be obtained by transforming one data set (e.g. counting the themes in a qualitative data set) in order to compare with the other data set (quantitative data set). Finally, the quantitative and qualitative results can be displayed and compared in tables or figures.
- *Connecting data:* sequential designs require that one data set is collected, analysed and interpreted before the next phase where the process is being repeated, which is when data are connected. Such information is used to inform subsequent data collection – for example, when the qualitative data (e.g. focus group) are used to develop the questionnaire to collect quantitative data, which in turn are calculated to obtain the results.
- *Embedding data:* this process refers to when one data set of secondary priority is embedded within a larger primary design. Examples of embedding data is when data are collected through questionnaires from a large group of participants, but from that group a smaller group is selected to participate in a focus group to provide answers to the research question. Qualitative data may precede an experimental trial to inform development of procedures. Alternatively, qualitative data may follow an experimental trial to help explain the results of the trial. In embedded designs, the data are being reported independently (Creswell et al., 2011).

From the integration process, several outcomes are possible in MMR (Brannen, 2016). There can be ***corroboration*** of the same results from both methods; ***elaboration*** where the qualitative data findings explain the quantitative findings; ***complementary outcomes*** where the results from the two strands may differ but when used together they provide new insights. In the case where there is a ***contradiction in the outcomes***, such as is the case in conflicting results, several strategies can be applied to explain (Moffatt, White, Mackintosh, & Howell, 2006). The researchers should either treat the methods as fundamentally different or explain the methodological rigour of each component. The data set can also be explored or additional data can be collected for comparative purposes. One can also explore whether the intervention worked or explore whether the outcomes of the quantitative and qualitative components match.

### Legitimising the research

**FIGURE 2:**
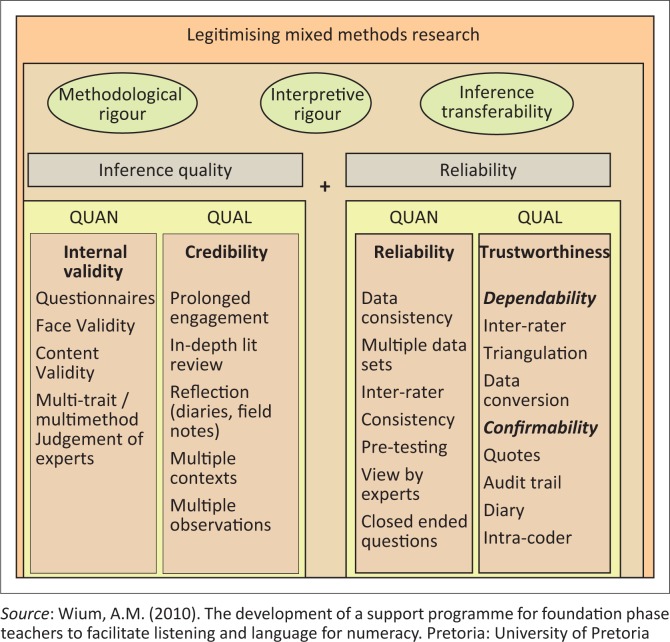
Aspects related to legitimising the research

The process of legitimising the research (which is the mixed-methods nomenclature for validity, reliability and trustworthiness) determines quality (Onwuegbuzie & Johnson, 2006) (refer to Figure 2). When critically appraising MMR, the legitimising process is a crucial step. Legitimising the research is the most important aspect of the research as findings that lack validity are considered of no use. Firstly, researchers should critically review the findings from both the qualitative and quantitative strands of the research (Garuth, 2013) and then proceed to assess how these findings have been integrated. In the discussion, the researcher should point out how the inferences relate to the objectives of the research.

Three processes determine the value of MMR, namely methodological rigour, interpretive rigour and inference transferability (refer to Figure 2).

The methodological rigour (also known as design quality) is concerned with the application of method and provides the standards for the assessment of MMR. Dellinger and Leech (2006) propose a validation framework consisting of concepts outside the more common terms. These terms include, for example, *foundational elements* (referring to prior knowledge of a construct or phenomenon under study that can influence the manner in which data are interpreted) and *inferential consistency* that depends on what is known from prior understandings, past research and theory, in addition to the study design, measurement and analyses.

The criteria for determining *quality* of the quantitative research are firstly to ascertain *validity*, which is to determine whether a measure actually measures what it is supposed to. Validity should be assured for each aspect of the research design, and only once validity has been established in both the quantitative and qualitative research, can the researchers consider this matter in MMR. Secondly, research has to show *reliability*, which is to measure and produce similar results across time and measures. Thirdly, it has to be *generalisable*, where findings should be applied across the wider population.

As a result of issues in meeting these stringent criteria, additional measures have to be introduced to ensure quality in qualitative research (Bryman, 2006). These include *credibility*, which ensures that findings makes sense and can be trusted. Secondly, the research findings must be *dependable*, which implies that they should be the same when repeated in a similar context. Thirdly, the research must also be *transparent* and findings must be *confirmed*. Lastly, the criterion of *transferability* should be met in that findings become relevant in other contexts.

Bryman (2006) discusses issues of *convergent criteria* (which is when the same criteria are being used for both the quantitative and qualitative components in different elements in the research), as well as *bespoke criteria*, where new criteria are instituted for mixed methods. When working within a pragmatic approach, one has to consider the purpose of the methods used, for example, in the case where qualitative methods are used to develop a questionnaire, it may be better to use convergent criteria (validity and reliability) rather than separate or bespoke criteria. Alternatively, when a qualitative project is preceded by a phase that collects quantitative data (e.g. demographic information), convergent criteria that support the trustworthiness of the research may be more suitable. Creswell and Plano Clark (2011) highlight additional potential validity issues that require attention:

- Inference quality, which refers to validity within a mixed-methods context, that allows the researcher to draw meaningful and accurate conclusions.
- Consequential validity, which is steered by the pragmatist approach as it guides the triangulation design which results in an overarching validity when researchers draw evidence from different sets of data that provide better results than any of the two data sets can do alone.

In MMR, it is important that each method is complete and meets the criteria for rigour as if it would when standing alone (Woolley, 2009; Irwin et al., 2014). An advantage of this standard is that each component can be published independently, particularly in sequential triangulation where projects are spanning over an extended period of time. The blending and merging of data is only conducted when results are fitted in a cohesive outcome or theory, or by confirming or revising existing theory. The critical appraisal of the research is considered an integral step in the MMR procedure.

## The mixed-methods research procedure

In MMR, researchers firstly have to determine whether a mixed-methods study is practical and what the justification for the mixing of methods is. This is followed by formulating the research questions and collecting and analysing the data. Lastly, the report has to be written (Creswell & Plano Clark, 2011). Several models for the development of MMR have been proposed (Leech & Onwuegbuzie, 2005; McMillan & Schumacher, 2006; Yin, 2006; Onwuegbuzie & Dickinson, 2007). For the purpose of this tutorial, the framework developed by Collins et al. (2006) consisting of four phases with 13 steps is considered as a practical guideline. Although several of the steps in this framework may at a glance resemble those used in mono-method studies, the elements within these steps are specific to MMR.

Researchers need to be cognisant of both the strengths or advantages and the challenges or controversies related to MMR that need to be considered.

## Critical review of mixed-methods research

### Advantages and strengths of mixed-methods research

Mixed-methods research maximises the strengths and reduces the limitations of single methods (Polit & Beck, 2010). When qualitative methods are used in combination with quantitative research, it provides a deeper insight into the context or the process and why treatment options are effective (Stewart & Richardson, 2004). Validity is increased when standard clinical measures are augmented through gathering the views of clients concerning treatment effectiveness (Kovarsky, 2008). The use of MMR can identify what to be mindful of in data collection or can identify the factors that impact on programme outcomes.

When quantitative and qualitative methods are used together, they both contribute to a common understanding of the research phenomenon (O’Cathain, Murphy, & Nicholl, 2008). The findings from one method can aid in the development of another, for example, findings from a focus group can determine the construction of a questionnaire. Mixed-methods research designs can include words, photos and narratives to supplement meaning to numbers, while numbers can add exactness to words, photos and narratives. A definite advantage of MMR is that it can be used to provide a voice to marginalised groups (e.g. when participants are not literate and need to be interviewed to obtain their views on a specific matter) (Teddlie & Tashakkori, 2003; Glogowska, 2011). Mixed-methods research findings are transferable, which is not the case when qualitative designs are used on their own. Qualitative research tends to be context specific because of the smaller sample size.

Despite these advantages of using MMR, there are several controversies and unresolved issues that researchers need to be cognisant of before embarking on such a venture. It is necessary to identify the potential threats or challenges that could have occurred during data collection and analysis, as it could impact on the validity of the findings. In order to enhance the quality of the MMR, the researchers should also indicate how such threats were countered by them (Venkatesh et al., 2013).

### Controversies and challenges in mixed-methods research

Controversies and challenges may occur in the conceptualisation stage of research (in providing a rationale for the mixing of methods), the operationalisation stage (how the methods will be used) or the synthesis stage where the findings of various methods used are interpreted. Teddlie and Tashakkori (2003) pointed to the following controversies which have also been experienced by some South African studies: (1) the nomenclature and basic definitions used in MMR remain to be disputed, (2) the use of MMR, (3) paradigms underpinning MMR, (4) issues related to the design of MMR (Schütte, 2016), (5) inferences derived from MMR (Wium, 2010) and (6) logistics when conducting MMR (Wium & Louw, 2011).

**TABLE 2:** The mixed-methods research process.

Phase	Steps
Phase 1: Formulation Phase	Step 1: Addresses the long-term aim of the study,
	Step 2: Objectives (e.g. to measure change; to understand complex phenomena; to test or generate new ideas; to inform constituencies; and to examine the past three goals).
Phase 2: Planning and Design	Step 3: Determines the research or mixing rationale that explains why the study is needed and why quantitative and qualitative approaches should be mixed. Collins et al. described four main rationales for MMR: (1) participant enrichment (e.g. when recruiting participants or to obtain participant feedback), (2) instrument fidelity, which assesses the suitability and use of research instrument and to validate individual scores on outcome measures, (3) treatment integrity (i.e. refining intervention implementation and the variables related with its context), and (4) significance enhancement (i.e. expanding the interpretation of the results and enhancing the interpretation of significant findings).
	Step 4: Consists of stating the mixing purpose, which explains what will be undertaken in the study and the purpose of mixing these two approaches. Collins et al. (2006) provide a long list of purposes for the mixing of methods, which have been grouped under each of the four rationales. Venkatesh, Brown and Bala (2013) presented seven purposes for MMR: Complementarity, which allows for mutual viewpoints about similar experiences or associations. This is to enhance and clarify the findings from one method with the results from another. Completeness, which is to confirm that there is total representation of experiences or that associations are reached. Developmental, which is to develop questions from one method that emerge from the inferences of a prior method or one method presents assumptions that can be tested in a subsequent method. One method informs the development of another method (e.g. interviews inform the development of a survey). Expansion, which is to explain and elaborate on the knowledge gained from a prior method. This adds breadth and scope to a project through the use of various methods for different components, or where one method could be nested within another method to provide insight into different levels of analyses. Corroboration or confirmation, which is to evaluate the trustworthiness of inferences gained from one method. Triangulation is convergence and corroboration of findings from different methods that study the same phenomenon (Morse, 1991). Corroboration or confirmation is used to evaluate the treatment integrity of a specific intervention through triangulation and corroboration. Compensation, which is to counter the flaws in one method by using the other. Diversity, which is to find contradictory or opposing viewpoints of the same experiences or associations (Venkatesh et al., 2013). The latter two steps particularly distinguish the MMR process from the mono-method processes, and therefore, mixed-methods researchers have to explicitly state the rationale and purpose for mixing quantitative and qualitative approaches.
	Step 5: Research questions guide the research as they determine the research design in terms of the stages and sequence of collecting the data (Onwuegbuzie & Leech, 2006). A MMR design should only be considered when it is called for by the research question as it may not be appropriate to answer all research problems. MMR simply is another option to be considered apart from the traditional quantitative or qualitative designs. It will be erroneous to select the design before the questions have been formulated. Firstly, the dominant nature of the research question needs to be determined – is it quantitative or qualitative? A general rule of thumb is that questions starting with the word ‘what?’ suggest a quantitative trend, and the word ‘how?’ implies a question that requires a more qualitative trend. Secondly, the part of the question(s) that is emphasised needs to be addressed more comprehensively, as that determines the research design. The research design in turn depends on the purpose for which the MMR design is intended.
Phase 3: Early development and pilot testing:	Step 6: Sampling design: Leech and Onwuegbuzie (2005) described four sampling designs: identical, where the same participants’ sample members participate in both the quantitative and qualitative components parallel, as the quantitative and qualitative samples are different but drawn from a common population nested (i.e. sample members selected for one phase of the study represent a subset of participants chosen for the other facet of the investigation) multilevel (i.e. using two or more sets of samples that are extracted from different levels of the study).
	Step 7: Mixed-Methods Design: The data from the quantitative and qualitative components are to be collected concurrently or sequentially. Data from the two components can also be collected partially or fully and can have equal or unequal status (Leech & Onwuegbuzie, 2005). In order to meet the outcomes of the study, specific attention should be paid to the methods for integration (e.g. during collection and analysis of the data). By triangulating data, sources convergence is sought across qualitative and quantitative methods. Key decisions to be considered are the level of interaction between the quantitative and qualitative strands, the priority of the quantitative and qualitative strands (weighting), the timing of the quantitative and qualitative strands and lastly the integration (where and how to mix quantitative and qualitative strands) (Irwin et al., 2014).
	Step 8: Early development and pilot study.
Phase 4: Advanced development	Step 9: Data collection: Data are collected, either simultaneously, concurrently or sequentially (Creswell & Plano Clark, 2011).
	Step 10: Data analysis: Data analyses can be carried out through either transformation, exploration of articles, and instrument development or examination of multiple levels.
	Step 11: Data validation: In the case of a sequential design (e.g. in the case where one cycle informs the design of data collection procedure in the second cycle), more data have to be collected, analysed and validated. After the validity has been established in both quantitative and qualitative strands of the research, the researcher has to consider the validity of the mixing process in the entire MMR study.
	Step 12: Interpretation: interpretation of the findings takes place only once all the data have been collected, analysed and validated. The goal of the interpretation phase is to make meta-inferences from combining quantitative and qualitative inferences (Teddlie & Tashakkori, 2003), which is specific to MMR as it is not common in mono-method studies. Researchers should interpret how the combined quantitative and qualitative approaches contributed to address the research problem and questions. It is necessary to specify whether the QUAL and QUAN results were merged, connected, embedded or mixed (Creswell et al., 2011).
Report writing	Step 13 consists of report writing, in which the researchers have to decide how to present the quantitative and qualitative components of the research. It is important to emphasise the contribution of the mixed-methods approach in the report (Creswell et al., 2011). It may become necessary for the researchers to reformulate the research question (Step 14), which in turn will set the research process in motion again.

These aforementioned controversies were later encapsulated in four challenges described by Onwuegbuzie and Dickinson (2007).

***Sampling issues*:** are inherent to both quantitative and qualitative research. In quantitative research, a significant threat to validity is the use of an *inadequate* or *inappropriate* sample (Schmidt, 1996). Lundie, Erasmus, Zsilavecz and Van der Linde (2014) experienced a limitation in the sample size of their study that developed a checklist of differential diagnosis of neurogenic stuttering. The use of non-random samples limits generalisation to other populations, as was noted in the study by Wium and Gerber (2016) that explored doctors’ knowledge of ototoxicity. The problem with adequacy should be countered by calculating the sample statistically. In qualitative research, threats related to *adequacy of the sample* are dealt with through data saturation. The challenge of representation (Denzin & Lincoln, 2005) that is often encountered by qualitative researchers also becomes an issue in MMR when researchers experience challenges in capturing the lived experiences using words and numbers. It may require representation of the total population (Irwin et al., 2014).

Another threat is appropriateness of the sample (Onwuegbuzie, Jiao, & Bostick, 2004), which can be countered by representation of the phenomena under study (e.g. how well the participants can articulate their experiences) (Irwin et al., 2014). Another sampling challenge is the merging of quantitative and qualitative research in sequential designs as researchers have to decide which results from the initial phase should be used in the follow-up phase. It may be challenging to select adequate sample sizes for both phases because of the unequal weighting thereof.

***Legitimation*:** it is more difficult to obtain legitimation in MMR than in either of the mono-method approaches. In quantitative research, the challenges experienced are in obtaining validity (e.g. construct validity, criterion validity and content validity) (American Educational Research Association, 1999). In qualitative research, researchers strive for credibility (internal validity), dependability (reliability), transferability (external validity) and confirmability (objectivity) of their findings (Denzin & Lincoln, 2005, p. 17). In MMR, it is expected of researchers to obtain credible, trustworthy, dependable, transferable and/or confirmable findings (Onwuegbuzie & Johnson, 2006).

***Integration*:** The third contentious issue in MMR relates to integration of quantitative and qualitative findings (Onwuegbuzie & Teddlie, 2003; Creswell et al., 2011; Glogowska, 2011). The unequal weighting of the two data sets may create a challenge in terms of validity (Irwin et al., 2014). It is about triangulating, expanding, comparing or consolidating findings obtained from data stemming from large, random samples in quantitative research with data obtained from the qualitative component that stems from small purposive samples. Another issue related to the integration of findings is when a decision has to be made in terms of integration during sequential designs. It may not be clear which results from the first phase will be the primary data in the second phase. In addition, researchers often make superficial claims to the use of MMR. Very often, questionnaires are mainly quantitative in nature (e.g. they not only consist mainly of closed-ended questions or checklists but also include a few open-ended questions), which cannot be regarded as true integration of methods. Such issues can be dealt with by rigorously defending the methodological choices. Researchers should also explicitly document methodological congruence.

Fourthly, there is a challenge concerning *politics*, based on the tensions that exist between researchers when combining quantitative and qualitative approaches. Methodological purists maintain that mixing is not possible from a paradigmatic perspective (Onwuegbuzie & Teddlie, 2003; Creswell et al., 2011; Glogowska, 2011). It may also be a challenge to convince stakeholders to value the findings from both the quantitative and qualitative components of the study (Irwin et al., 2014).

*Other challenges* may be encountered when conducting MMR (Morse, 1991; Creswell et al., 2011; Glogowska, 2011). When the quantitative and qualitative results do not confirm each other, it is best to report both sets of findings to emphasise the complexity of the phenomenon or intervention, which is often the case in health and education contexts. In such a case, the use of MMR is particularly useful, as by doing so one can demonstrate how mixed methods can tell a more comprehensive and realistic story, for example, about what works under which circumstances and with whom.

Many MMR projects end up being published separately as mono-method research. Reasons for this may be that the authors expect readers to have an interest in one specific aspect of the study or because of the strict page and word limitations in some journals. Mixed-methods research is longer than mono-methods research, and researchers may find such limitations challenging to get their work published. In sequential studies, the timing of the various components of the research may also be the cause for not publishing mixed-methods studies as researchers may feel pressured to publish results obtained from earlier phases first.

There may also be logistical challenges, which are related to availability of resources (Onwuegbuzie & Teddlie, 2003; Creswell et al., 2011; Glogowska, 2011), as MMR is more costly as a result of more than one type of data collection procedure being used. Research training may be required as the researchers need to be tri-skilled in quantitative, qualitative and MMR (Irwin et al., 2014). Mixed-methods research requires greater effort than single-method designs and can pose a challenge to a single researcher, especially in the case of concurrent designs. In the case of sequential designs, it may be more time consuming. It may be best to work in a team (Creswell et al., 2011).

Researchers should, however, not be discouraged by these challenges but should familiarise themselves with the MMR literature and explore the exciting possibilities that mixed-methods designs can offer. The challenges can, however, be turned into advantages as it requires researchers to be versatile and to work in teams and across disciplines. Interprofessional collaborative practice in both health care and education settings is supported across the globe (Garuth, 2013). The mixed-methods approach is ideally suited for research in inter-profesional collaborations to improve outcomes for individuals with communication disorders and their families and to deliver the highest quality of care across settings.

## Conclusion

The MMR approach offers an exciting avenue for exploring multidimensional and complex questions in the disciplines of speech-language therapy and audiology. Speech-language therapists and audiologists serve clients with complex conditions such as speech, language, hearing, balance and swallowing problems. These clients are affected by the physical, social and attitudinal environments in which they live (World Health Organization, 2001). Speech-language therapists and audiologists are required to provide EBP services, which are multifaceted to meet the needs of these individuals and their significant others.

The therapeutic process itself can be viewed as a MMR process, as assessment procedures consist of quantitative data that are complemented by qualitative data obtained from interviews with the clients and their families. A dearth of knowledge continues to exist regarding the most efficacious intervention approaches for clients with a variety of communication disorders.

Interprofessional research is becoming increasingly popular and lends itself to MMR. Researchers working in teams can draw on the strengths of different disciples and their research approaches. Such collaborative enterprises will contribute to capacity building. Researchers, speech-language therapists and audiologists are encouraged to make use of MMR to address the complex research issues in the multicultural, multifaceted South African context. Mixed-methods research makes an important contribution to the understanding of individuals with communication disorders, and in turn, researchers in the two disciplinary fields of speech-language therapy and audiology can contribute to the development of this research approach. Mixed-methods research is well suited to the complexity of South African contexts and its populations, as it can provide multiple perspectives on a topic.
